# The Learning Environment of Student Nurses During Clinical Placement: A Qualitative Case Study of a Student-Dense Ward

**DOI:** 10.1177/23779608211052357

**Published:** 2021-10-27

**Authors:** Siri Vestby Bøe, Jonas Debesay

**Affiliations:** 60499 OsloMet – Oslo Metropolitan University, Department of Nursing and Health Promotion, Postboks 4, St. Olavs plass, 0130 Oslo

**Keywords:** educational models, nursing baccalaureate, learning, qualitative research, nursing education research, clinical placement, learning environment, clinical supervision, confidence

## Abstract

**Introduction:**

Ensuring the quality of clinical placements has long been a challenge in nursing education. This is partly due to a growing aging population requiring health services, and an increased need for nursing workforce. Both in Norway and internationally, there is a rise in the use of student-dense models, wherein several students are placed together on the same ward at the same time where the supervision of the students is the collective responsibility of the nurses.

**Objective:**

The aim of this study was to explore factors that promote or inhibit learning in a student-dense ward when used as a model for clinical placement in hospitals. We examined how clinical placement is experienced in a student-dense ward, as well as how learning is facilitated.

**Methods:**

A qualitative case study design was used to capture the learning environment on the student-dense ward in a comprehensive way**.** We used focus group interviews, in-depth interviews, and observations with students and employees at a major hospital in Norway.

**Results:**

Our findings showed that the orientation days and the teaching activities in student-dense wards, the feedback students receive, the clinical facilitator's role and the student community were factors that had particular importance for good learning environments in this placement model.

**Conclusions:**

To ensure the quality of clinical placements, more attention should be paid to these factors in the planning, organization, and facilitation of new and existing student-dense wards. It is paramount to provide students with thorough written feedback and to secure the clinical facilitators with enough time to conduct student supervision when organizing clinical placement as student-dense wards.

## Introduction

Clinical placement is an essential part of nursing education which educational institutions spend significant resources to organize (Forber et al., 2016; Hooper et al., 2020; Jayasekara et al., 2018). Forber et al. (2016) point out that the effective implementation of models for clinical education is vital for students’ learning outcomes. At the same time, research shows that the facilitation of good and relevant clinical placements by both educational institutions and health institutions remains insufficient (Budgen & Gamroth, 2008; Croxon & Maginnis, 2009; Ekstedt et al., 2019).

As the population grows older and the need for healthcare increases, more nurses are being educated than ever before. In Norway, this trend has led to pressure on clinical placement providers to the point that it has become challenging to offer sufficiently relevant clinical placements (Hjemås et al., 2019; Ministry of Education & Research, 2011–2012). To provide the growing number of nursing students with good quality placements, educational institutions and health institutions are increasingly applying new models to clinical education. These models offer the possibility for more students to undergo their clinical placement at the same time and in hospital wards where all the nurses are made responsible for their supervision. One model goes by the name “student-dense ward” in Norway (Halse et al., 2016; Halse & Hage, 2004; Strand et al., 2013). Similar models have been launched in other countries under different names, for example, dedicated education unit (DEU) (Hooper et al., 2020; Pedregosa et al., 2020; Pedregosa et al., 2021), clinical education unit (CEU) (Jayasekara et al., 2018; Pedregosa et al., 2020), the cluster model (Bourgeois et al., 2011; Croxon & Maginnis, 2009), and collaborative learning unit (CLU) (Marcellus et al., 2019).

## Review of Literature

Research shows that organizing clinical placement as student-dense ward models can positively influence students’ professional development (Halse & Hage, 2006; Hooper et al., 2020) and provide satisfactory learning outcomes (Halse et al., 2016). Moreover, features of the model such as students taking responsibility for their own learning, working with different nurses, and being included in a learning environment where they have the attention and support of the nurses has a positive impact on the students’ learning process (Bourgeois et al., 2011; Budgen & Gamroth, 2008; Hooper et al., 2020). Distinctive features of the student-dense ward model include the following: much of the learning happens between students, students observe how different nurses work, and there are high demands placed on the students to take responsibility for their own learning. In addition, most student-dense wards arrange orientation days, including teaching activities, at the beginning of the clinical placement period (Bjerknes & Christensen, 2015; Halse et al., 2016; Halse & Hage, 2004; Strand et al., 2013).

The students’ learning environment is central in clinical placement. A supportive environment with planned learning activities is essential (Vinales, 2015). For example, Ekstedt et al. (2019) show that a good learning environment in clinical education depends on engagement and collaboration between supervisors and teachers as well as good arrangements for welcoming students. Therefore, the clinical learning environment affects learning outcomes and significantly impacts on both the clinical learning experience and students’ satisfaction with the nursing profession. In addition, the interaction and psychosocial factors, organizational culture, and teaching and learning components are characteristics of the clinical learning environment that may have an impact on the students’ students’ experience of learning in clinical placement (Flott & Linden, 2016).

According to Hammer in Eriksen (2006), confidence is related to a person's abilities to predict, prepare for, and control an event. If students feel confident in the clinic, they will dare to be proactive in their learning, utilize their own resources, and work deliberately to achieve their learning goals. To feel belonging and support in the clinical setting is also crucial for students’ confidence and learning (Holmsen, 2010). Although the importance of confidence and well-being for students’ learning in a student-dense ward has scarcely been explored, several studies have evaluated the model's general viability and feasibility (Bourgeois et al., 2011; Crawford et al., 2018; Halse & Hage, 2004; Halse & Hage, 2006; Rhodes et al., 2012). Both Crawford et al. (2018) and Halse and Hage (2006) evaluated the organizing of clinical placement as student-dense wards, considering whether the model meets students’ needs for relevant learning situations. Moreover, Halse et al. (2016) examined how the various participants in student-dense wards perceive students’ learning and the practical implementation of the model.

Several studies have also compared student-dense ward models with more traditional clinical education models (Croxon & Maginnis, 2009; Pedregosa et al., 2020). For example, Pedregosa et al. (2020) explored how well DEU and CEU, among other models, facilitate nursing students’ students’ learning compared to traditional models. They found that student-dense ward models provided results that are at least as good as traditional one-to-one mentoring models. Nevertheless, students may also feel insecure when several nurses supervise them at the same time in a student-dense ward, while the nurses may often find that they have too little time to spend with students, given that they constantly must prioritize patient-oriented work over the responsibility of student supervision (Rhodes et al., 2012). Thus, the aim of this study is to explore factors that promote or inhibit learning in a student-dense ward when used as a model for clinical placement in hospitals. Since the student-dense ward is a key measure to increase the capacity of nurse education, it is important to explore how the model can contribute to securing good clinical education.

## Methods

In this descriptive case study, we explore students’ and key participants’ experiences, actions and learning activities as well as the learning environment in a specific student-dense ward. The case study design was chosen to allow the deployment of several qualitative methods for data generation, identify several aspects of the study object and capture the learning environment on the student-dense ward in a more comprehensive way (Stake, 1995). The research question was: What factors promote or inhibit learning in student-dense ward as a model for clinical placement in hospitals? To enable a rich, detailed, and in-depth understanding of the phenomenon, we chose to focus on only one student-dense ward as a single case. Forrest-Lawrence (2019) notes that the single-case design searches for an in-depth understanding of one particular case and enables a richer understanding of the studied issue.

### Setting

The study was conducted at a student-dense surgical ward with 22 patient beds at a major university hospital in Norway. This ward has organized its clinical placement activities within the student-dense ward model for more than ten years. Four times a year, the department welcomes eight students for an eight- or ten-week clinical placement. The day-to-day supervision of each student is performed by a mentoring team of three to four nurses. The department has two clinical facilitators who are formally responsible for supervision. They combine this responsibility with three-shift rotation and patient-oriented work.

The clinical facilitators offer two orientation days with informational and teaching activities when the students arrive on the ward. The students are introduced to the most common learning situations, routines on the ward, and the diagnoses and procedures they are likely to encounter. During the orientation days, students and clinical facilitators also share mutual expectations for the clinical mentorship.

Lessons in practical nursing procedures (PP lessons) are specific for this particular student-dense ward. These lessons are held weekly by the ward's nurses and are developed so that the students and nurses may devote more attention to practical nursing procedures. The purpose of the PP lessons is for students to practice procedures under experienced nurses’ theoretical and practical guidance.

The use of evaluation forms is an essential part of the student-dense ward model. The students evaluate their efforts, learning outcomes and further learning needs daily by filling in the evaluation form. The supervisor then gives a general assessment based on the school's assessment criteria. All assessment items are on the Corrected “as expected”, or “above expected”. On this form, the supervisors also provide written feedback after each shift on the student's achievement of learning outcomes. The student's overall performance in the clinic is formally assessed twice during the eight weeks of practice in an intermediate and final evaluation. This takes place in meetings including the student, the clinical facilitator, and a nursing educator from the university. At the meeting, the student is given the opportunity to reflect on the learning outcomes set at the beginning of the practice term.

### Data Collection

Researchers recruited a purposeful sample of participants for the study (Creswell & Poth, 2018; Stake, 1995) (see Table 1). They were recruited either because they were students in clinical placement or worked on the student-dense ward. The participants had different roles in the ward, which means that data from several sources were collected to see whether the case being studied constitutes the same meaning under different circumstances (Stake, 1995). This study is based on two focus group interviews, three in-depth interviews, and observations with nursing students and other key participants in the relevant student-dense ward (see Table 1). Focus group interviews were chosen as interview method with the student participants because they constituted a homogeneous group from the same environment. We invited all eight students, and the same student group were interviewed twice during their clinical placement to capture whether students’ experiences changed during the placement period. In addition, researchers conducted in-depth interviews with the Head of the section, one clinical facilitator and one nurse from a mentoring team. These participants had different affiliations and knowledge about the students and the student-dense ward. The first author conducted all the interviews and observations. She is a registered nurse and has extensive experience in student supervision from another student-dense ward.

**Table 1.** Overview of Participants and Data Collection.

Total participants	Focus group interviews (FG)	In-depth interviews	Observations Duration in total: 13 h
**Students (N = 8)**	FG 1 (N = 7) Duration: 1 h FG 2 (N = 6) Duration: 1 h 20 min		Preparatory clinical meeting (N = 6) Duration: 1 h Orientation days (N = 8) Duration: 6 h
**Clinical facilitators (N = 2)**		(N = 1) Duration: 40 min	Orientation days (N = 2)
**Nurse from a mentoring team (N = 1)**		(N = 1) Duration: 45 min	
**Head of Section (N = 1)**		(N = 1) Duration: 30 min	
**Nurse educator (N = 1)**			Preparatory clinical meeting (N = 1)

A semi-structured interview guide with open-ended questions was used during all the interviews. Examples of questions from the interview guide include: “Can you describe what is important for you to learn in a student-dense “ward” (focus group interview 1), “What opportunities and challenges do you think the students face in a student-dense ward?” (in-depth interviews), and “What in a student-dense ward affects your experience of safety/insecurity? “(focus group interviews 1 and 2). Roles, relations, and communications between the participants in the student-dense ward was in focus during the observations. Examples of observational questions include: “What are the learning situations? Are these relevant/appropriate?”, “Do the students seem confident/nervous?”, “How is their relation to the clinical facilitators?” and “How is the communication between the clinical facilitators and the students?”.

### Data Analysis

The data consisted of transcribed interview material and field notes. The interviews were audiotaped and transcribed verbatim. The field notes were completed immediately after the observations while the observations were fresh in memory, and the audio recordings were transcribed close to each interview. The data was analyzed using a six-step thematic analysis approach (Braun & Clarke, 2006). We chose this analysis method because of its flexibility and inclusive approach, regardless of data source (Braun & Clarke, 2006). The data analysis had an inductive, data-driven approach, where the themes emerged in the course of the analyzing process. The analysis followed an iterative process in which codes, sub-themes, and main themes were processed and revised in several phases (see Table 2). The analysis tool NVivo 12 was used in the coding process.

**Table 2.** The Data Analysis Process.

Quotations from participants	Codes	Sub-themes	Main themes
*I think it was good to get some insight into what will happen when and get to know a bit about the ward before we started. It makes it a little easier to come when you already have two familiar faces from the first day and so on [Tine, student]*. * *	A good welcome	The orientation days contribute to confidence and learning	Valuable orientation days and practical procedures lessons
*Many of the nurses I’m with have said just put it [the evaluation form] on my shelf. I won’t look at it until the end of the [placement] period anyway, but then I won’t get anything back for it. If I don’t get feedback until the end of my placement, I don’t know what to do [Live, student].*	Written feedback	Attitudes towards the written feedback	Unstructured and random written feedback from supervisors
*No, I feel that way too. I still haven’t been with any of them [the clinical facilitators] yet, and now it's only a bit more than a week until mid-term assessment. And even though we fill in those forms, I don’t feel like I’m getting any evaluation. I feel like I can’t show my skills, I can itemize what I’ve been through, but they don’t get a proper picture of me. Anyway, you don’t get to show yourself properly. So, I, yes, I’m a bit tense about that mid-term assessment. I think it's a little weird [Live, student].*	Uncertainty about the assessment basis	The clinical facilitators have little time for student work	Clinical facilitator's role and predictability of the supervision for students
*Security is being with other students. You chat with them. Listen to how they experience things. You use other students for reassurance and ask the questions that perhaps, after seven weeks, are a little too stupid [Atle, student].*	Lowering the bar for asking fellow students stupid questions	Fellow students mean increased confidence	The benefits of the co-location of many students on the student-dense ward

## Results

The participants consisted of five groups: eight students, one Head of section, two clinical facilitators, one nurse educator from the university and one nurse from one of the mentoring teams. The student group age ranged between 20 and 35 years and consisted of seven females and one male. The other participants were between 25 and 65 years old. The students were in the third and final academic year of their bachelor's degree program in nursing, and this was their first clinical placement in a hospital ward. Although the clinical facilitators were charged with the overall responsibility of supervision, it was the ward's separate mentoring teams who provided the day-to-day supervision of the eight students.

Factors that particularly affected student learning on the student-dense ward are presented according to four themes 1) valuable orientation days and practical procedures lessons, 2) unstructured and random written feedback from supervisors, 3) the clinical facilitators’ role and predictability during supervision, and 4) the benefits of the co-location of many students on the student-dense ward. All participants’ names presented here are pseudonyms.

### Valuable Orientation Days and Practical Procedures Lessons

One of the aspects that students greatly appreciated about the student-dense ward was the planned orientation days. They experienced these days as educational and relevant time spent with engaged and knowledgeable clinical facilitators. They were welcomed by the facilitators, who wanted them to feel at home and learn in a safe environment. For example, before they met the students on the first day, the clinical facilitators said to each other: “A lot of things are frightening for them [the students], so we shouldn’t be frightening as well.” The following quote from student Alice indicates that the orientation days helped to calm otherwise nervous and excited students: “Now I feel safe. They’re nice, and I’m sure this is going to be all right.”

The weekly practical nursing procedures lessons (PP lessons) were received particularly positively by both students and nurses. Here, students learned from their supervisors through specific and relevant teaching, and they could practice procedures in small groups in a safe environment, as exemplified in this quote:

*The fact that they’ve arranged for us to have some lessons while we are here [in placement] feels very good. I think it's good to get some theoretical teaching because, otherwise, it depends on which supervisor you have. Like Anine [fellow student], perhaps she has the greatest on wounds in the department as a supervisor. So, she has probably learned a lot more about wounds than, for example, me. So, when we had a PP session about wounds, I learned more in that lesson than I’ve done during the whole clinical education* [Erika, student].

Both the students and the staff considered having PP lessons useful. In addition to the benefits for the students, the Head of Section, Tove, also talked about the benefits for the staff: “They have to engage in evidence-based learning, which is grounded in the literature and is completely updated. They get to stay up to date. It's a win-win situation.” One of the nurses from the mentoring team, Tuva, found it rewarding to teach PP lessons. She was able to update her knowledge and found it “really fun to teach when you notice that students learn something.” When the students were engaged, and once she had developed an effective and dynamic teaching relationship with the student group, she experienced that both she and the students benefited from the lessons.

### Unstructured and Random Written Feedback From Supervisors

The students experienced that the written feedback from nurses in the mentoring team was of varying value for their learning outcomes and progression through the clinical placement. Although they received some thorough feedback that was instructive for their learning process, the students expressed that they received too little of it. However, most experienced a satisfactory amount of oral feedback after they had performed practical procedures. This quote summarizes many of the students’ experiences with written feedback on the student-dense ward:

*We don’t get too much feedback, but I think it's been good after every procedure. At least on the oral part. I have received proper [written] feedback once, and I found the feedback to be very instructive (…) I really want to know what I can do better. Getting constructive feedback, both positive and negative. I miss a bit of that* [Anine, student].

Often, the written feedback gave the impression that the supervisors “write just to write,” or the students received the feedback a long time after the relevant shift, which meant that they were left not knowing where they needed to improve. Many of the students made statements such as this one by Live: “If I don’t get feedback until the end of my clinical placement, I won’t know what to do.” When the supervisors delayed providing feedback, they tended not to remember for which shift they were supposed to provide feedback.

Anine experienced feeling “very small” after being told that feedback on the evaluation forms was not “given priority during a busy day at work” when she requested feedback. Moreover, the students experienced that not even the clinical facilitators always gave priority to the evaluation forms. During the focus group interviews with the student participants, it emerged that those who had received helpful feedback throughout the clinical placement period felt more secure than those who had received less feedback, as this quote from Line might indicate: “When I know that I have received feedback in the past (…) received something that reassures me a little, then it has helped later in the period.”

How much helpful written feedback the students received varied depending on the supervisor. Several students said that they had experienced a gap between what the student-dense ward promised and how much feedback they received.

### Clinical Facilitator's Role and Predictability for Students During the Supervision

According to observations, the clinical facilitators sincerely wanted to reassure the students. They wanted to be good role models and allow the students to “lower their shoulders” so that they could focus on learning. The clinical facilitators focused on conveying security and ensuring that the knowledge they possessed would be shared in such a way that “students can see themselves in the same role” as a nurse. Moreover, they viewed the facilitators’ role as one of conveying confidence and knowledge:

*As a supervisor, as a person with authority, saying “No, it was my fault” or “I shouldn’t have done it” helps to lower their [the students’] shoulders, I think. Because I believe a supervisor is someone who shows a path. It's not necessarily someone who does everything right. Okay, you have a lot of knowledge, but then the aim is to share it so that students can see themselves in the same role. So that they can say, “I can’t wait to be like that,” for example, or “That's not how I want to be.” Yes, convey security* [Kari, clinical facilitator].

The clinical facilitators were often assigned little time for the supervision of students. Consequently, many of the students rarely saw the facilitators and felt that they did not prioritize seeking contact with them. The facilitators had to combine supervision and follow-up with students with their patient-related work and shift schedule. The shift schedule also meant that in some periods, for example, after night shifts, they had time off, during which they were not available for students.

The fact that students felt that they had limited contact with the clinical facilitators made some of them insecure about mid-term and final assessments. They felt uncertain about whether the facilitators had a good enough basis for assessment and were not confident that the facilitators had communicated enough with the nurses who supervised them daily. The students did not always feel that they had demonstrated their skills and abilities through what they wrote on the day-to-day evaluation forms, especially when they had not had supervision from the clinical facilitators themselves. Those who had been supervised by the clinical facilitators during a shift or two felt more secure about the mid-term and final assessments. Therefore, it seemed that the facilitators contributed positively to students’ overall learning on the ward, but they did not obtain a complete overview, which could have given the supervision more predictability for the students.

### The Benefits of the co-Location of Many Students on the Student-Dense Ward

The focus group interviews clearly showed that the co-location of several students on the same ward had many advantages. The most prominent was how fellowship contributed to students’ sense of safety and that they could learn from and with each other. For example, one student said that it was reassuring to make clinical decisions with another person. The threshold for asking questions to fellow students was lower than for asking the supervisors about what the students considered minor issues. Here is how a student described an episode when she could not figure out how the decontaminator worked:

*I saw that Erika [fellow student] was in a patient room, and I speculated about how that bedpan, which way it was going in that thing [the decontaminator]. Then I stopped and waited outside the door for Erika to come out, so I didn’t have to go to the duty room and ask my supervisor. It always helps to have someone you know a little better nearby* [Line, student].

Especially when it was quiet on the ward, students felt it was valuable that there were several of them on a shift together. This allowed them to practice procedures, such as placing peripheral venous catheters on each other, examining equipment, and teaching each other what the equipment is used for and how it works. They could also join a fellow student when he or she performed a procedure on a patient. This way, the students filled quieter periods with educational activities in fellowship.

The students tried out additional collegial collaboration by teaming up in pairs on the night shifts. This allowed them to collaborate on patient care and to benefit from each other's knowledge, as this quote indicates:

*Then we managed to take responsibility for that patient alone because we covered each other's knowledge gaps. We could say that we could manage this [patient] without help from the nurse because, together, we had enough knowledge to do it* [Erika, student].

The students found it reassuring to be paired up when making decisions. They also felt that they could take more responsibility with a fellow student than alone with a supervisor in what they perceived as a safe environment.

## Discussion

The purpose of this study was to explore factors that promote or inhibit learning in a student-dense ward as a model for clinical education in hospitals. The key findings revolve around the good reception students experienced at the ward, the varying forms of written feedback from the supervisors, the vital role of the clinical facilitators, and the student fellowship that gave students the perception of a safe learning environment on the student-dense ward. Although the students mainly experienced the clinical placement as positive, it also seemed that the ward had set some unrealistic expectations for follow-up of the students and the a mount of feedback on the evaluation form.

Among the learning activities that the students appreciated most were the orientation days, which greatly contributed to their learning and confidence-building. A study by Holmsen (2010) shows that the first few days in clinical placement can be perceived as overwhelming by student nurses; therefore, it is essential for them to be overseen and looked after by the staff. Information about what they will face in the clinical placement period, what to expect of learning opportunities, and what is expected of them as students are important components that build their confidence (Holmsen, 2010). These components were integrated into the orientation days on the student-dense ward we have studied, and the students articulated how it contributed to their feeling of confidence. Similarly, Maasø (2016) has examined the quality of clinical placements and points out that students are anxious before they start clinical education, and a good reception on their first day has considerable implications for the entire learning process. To ensure a good reception, it is highly recommended that the supervisor be well prepared so that students feel expected and welcome on the ward. This first impression is often something students take with them throughout the clinical placement period (Maasø, 2016). In the present study, the students experienced a caring reception, especially from the clinical facilitators, which helped them feel less nervous and more prepared for the clinical education.

Lessons of practical nursing procedures (PP lessons), arranged weekly, were considered central to learning on the student-dense ward and provided the students with relevant teaching activities in a safe environment. These classes were also appreciated by the nurses who taught the classes. In accordance with our findings, studies show that the best way to be introduced to a new procedure is to divide students into smaller groups, which makes it easier to observe the demonstration, practice the procedure, and be active in the learning process. It is also an advantage if the same person who teaches the practical procedures also supervises them in their performance during the clinical placement (Muthathi et al., 2017).

One of our main findings was that during the placement period, the students experienced inadequate written feedback on their evaluation forms. Previous research on models where multiple nurses shared the responsibility of supervision indicates that students, clinical facilitators, and nurse educators may experience challenges in obtaining information about students’ performance and progress in practice (Budgen & Gamroth, 2008; Grealish et al., 2018; Halse et al., 2016). In the current study, no evidence was found that the clinical facilitators or the nurse educators experienced challenges in obtaining information from the mentoring teams. One reason for this may be that the organization of student-dense wards requires close cooperation between the clinical facilitator and the mentoring team, which ensures sufficient exchange of information both orally and through the students’ daily evaluation forms. The students’ insecurity might therefore have more to do with their need for continuous and predictable evaluation throughout the practicum and not only during the mid- and end-term evaluations.

The studies conducted by Adamson et al. (2018) and Hattie and Timperley (2007), both of which dealt with feedback in clinical placement, found that students had to actively request feedback to take full advantage of the placement period. However, in many cases, the student viewed feedback as someone else's responsibility to provide (Hattie & Timperley, 2007). The students experienced the supervisors’ attitudes towards the evaluation form varied and that written feedback was not given priority. Although some students commented that they had requested feedback, it is uncertain how much feedback they sought from the supervisors. At the same time, Hattie and Timperley (2007) noted that there must be an open and trusting relationship between the student and supervisors for students to feel that they can request feedback. This necessarily becomes more demanding when students must engage with each member in the mentoring team on a student-dense ward and there is less time to get to know and build trust with those who will evaluate and assess them. Therefore, the sharing of the evaluation of students among several nurses seems to be a weak spot of the student-dense ward model and should be strengthened to contribute to a good learning environment for students.

Continuous feedback from supervisors is essential for nursing students’ learning in clinical placement (Giles et al., 2014; Hattie & Timperley, 2007; Holmsen, 2010; Jansson & Ene, 2016; Strand et al., 2013; Sweet & Broadbent, 2017). Feedback on the evaluation form gives students specific feedback and thus an overview of what they need to focus on in future work (Strand et al., 2013). Continuous and specific feedback during the placement period contributes to the student's confidence and provides knowledge regarding where they stand in the learning process (Holmsen, 2010). On the other hand, inadequate feedback from supervisors may inhibit learning (Giles et al., 2014; Sweet & Broadbent, 2017). In our study, the written feedback from supervisors was perceived as particularly important for students to ensure predictability as well as for their sense of confidence. At the same time, the quantity and quality of feedback from the various supervisors were insufficient. To ensure the progression of learning, continuity, and the fair evaluation of students when they are supervised by different supervisors, it may be appropriate that feedback through the evaluation form, one of the core elements of a student-dense ward, be given priority.

The clinical facilitators in our study were forced to switch between patient-related work and student-related work. This is not uncommon since the amount of time that facilitators allocate to following up on students might differ from one student-dense ward to another (Halse et al., 2016). In Croxon & Maginnis's (2009) evaluation of clinical education models, the role, the resource, and support of the clinical facilitator were the most positive results of “the cluster model.” The students in the study found that the clinical facilitators were available to both students and supervisors by facilitating and overseeing accessible learning situations, supervising students during nursing procedures, relieving the day-to-day supervisors, and being available to share their knowledge of the ward. Bourgeois et al. (2011) study shows that a prerequisite for success in the DEU clinical education model is the clinical facilitator's support for nurses in their daily supervisory responsibilities and collaboration with them. In this light, it seems to follow that the more time clinical facilitators—key individuals in the implementation of a student-dense ward—have allocated to student-related work, the better it is for the students’ confidence in relation to evaluation and assessment and their learning process. This suggests that the role of clinical facilitators should be strengthened to ensure high-quality supervision, follow-up, and evaluation of students in student-dense wards.

One central finding was that students learned from and with each other when several students were placed in the same ward and had the opportunity to take care of patients together. This is consistent with previous studies, which also found that working together to provide patient care was positive for the students’ learning process and sense of confidence (Bourgeois et al., 2011; Jansson & Ene, 2016; Pålsson et al., 2021; Stenberg et al., 2020; Strand et al., 2013) and for their experience of self-efficacy (Pålsson et al., 2017). To jointly take responsibility for nursing a patient increases students’ learning outcomes due to the organization, planning, and practice of nursing together (Pålsson et al., 2021). Working in a group allows them to share workloads, discuss experiences and challenges, and validate their practice (Bourgeois et al., 2011). The opportunities to help each other in the learning process when there are many students in the same location seem like one of the most valuable characteristics of the student-dense ward model (Halse & Hage, 2004). Stenberg et al. (2020) discovered that supervisors found that students who had been in a clinical placement where collaboration with fellow students was central (peer-learning model) developed at a professionally higher level as they were more reflective and analytical. When we look at our positive findings regarding collaboration with fellow students in pairs with previous peer-learning research, they suggest that this type of collaboration positively impacts the students’ learning process and confidence in student-dense wards.

## Strengths and Limitations

In this single-case design we have used data triangulation and methodological triangulation. For example, we have included participants with different relations to the student-dense ward, and we have used different methods for data collection. Creswell and Poth (2018) states that the triangulation helps confirm results and strengthens the validity of the study.

The first author's prior knowledge with the student-dense ward model may have been an obstacle to sufficient openness to impressions and new knowledge, as previous knowledge may lead to taking some issues for granted. At the same time, an in-depth knowledge of the field may have provided an opportunity to ask relevant research questions and timely follow-up questions during the interviews. Nevertheless, the first author has been reflexive in the way she has challenged own preconceptions by discussing analyses and findings with the second author. The first author has carried out data collection, analysis, and interpretation, which may have affected the interpretation of the data. To contribute to nuanced interpretations of the data material, the second author assisted and validated the analysis process throughout.

The observations have taken place during the introductory activities. Observations in the clinic may have provided access to richer data about the students’ situation in student-dense ward.

As a single-case study, the data collection was undertaken in only one ward at one hospital. Therefore, the transferability to other student-dense ward models may be limited. The student-dense ward model has been used for some time in this particular hospital ward, and therefore it is a limitation to transferring the study results to another location with less experience.

It is also probable that the ongoing COVID-19 pandemic made the challenges experienced by the students clearer or more difficult since health institutions and society at large have been subject to immense limitations as they strive to maintain infection control and simultaneously allow clinical placement.

## Implications for Practice

Increased use of the student-dense ward model may improve the capacity and quality challenges experienced by nursing education by offering more students relevant clinical placements. Clinical facilitators and mentoring teams must be given sufficient training and time in order to improve their follow up of a group of students during clinical placement.

## Conclusions

In this article, we found that certain factors especially inhibit or promote learning using the student-dense ward model; this suggests that there should be a greater focus on those factors in planning, organizing, and facilitating new and existing student-dense ward models. The orientation days, the teaching on the student-dense ward, and the fellowship between the students had particularly positive impacts on the learning environment. At the same time, there were a number of challenges with regard to structuring the feedback and the organization of the clinical facilitator's role, which meant that the students’ well-being and learning on the student-dense ward could have been better. Although the aspirations associated with the operation of the student-dense ward were high, they were not always accompanied by specific measures. To ensure the quality of the model, it is important to secure thorough written feedback for the students, allow time for the clinical facilitators to follow them up, and acknowledge the benefits of student fellowship during clinical placement.

## Ethical Approval and Informed Consent

The study has been approved by the Norwegian Centre for Research Data (NSD) (2021) (ref. no.: 546799) and the local data protection officer at the hospital where the study was conducted. The participants gave their informed written consent before the data collection started. They were also informed that they could withdraw from the study at any time.

The study's objective falls outside the scope of the Health Research Act (Ministry of Health & Care Services, 2021) and did not require additional approval from the Regional Committee for Medical and Health Research Ethics (REK) (2021).

## Supplemental Material

sj-docx-1-son-10.1177_23779608211052357 - Supplemental material for The Learning Environment of Student Nurses During Clinical Placement: A Qualitative Case Study of a Student-Dense Ward Click here for additional data file. Supplemental material, sj-docx-1-son-10.1177_23779608211052357 for The Learning Environment of Student Nurses During Clinical Placement: A Qualitative Case Study of a Student-Dense Ward by Siri Vestby Bøe and Jonas Debesay in SAGE Open Nursing
